# Lenalidomide and dexamethasone with or without clarithromycin in patients with multiple myeloma ineligible for autologous transplant: a randomized trial

**DOI:** 10.1038/s41408-021-00490-8

**Published:** 2021-05-21

**Authors:** Noemi Puig, Miguel T. Hernández, Laura Rosiñol, Esther González, Felipe de Arriba, Albert Oriol, Verónica González-Calle, Fernando Escalante, Javier de la Rubia, Mercedes Gironella, Rafael Ríos, Ricarda García-Sánchez, José M. Arguiñano, Adrián Alegre, Jesús Martín, Norma. C. Gutiérrez, María J. Calasanz, María L. Martín, María del Carmen Couto, María Casanova, Mario Arnao, Ernesto Pérez-Persona, Sebastián Garzón, Marta S. González, Guillermo Martín-Sánchez, Enrique M. Ocio, Morton Coleman, Cristina Encinas, Ana M. Vale, Ana I. Teruel, María Cortés-Rodríguez, Bruno Paiva, M. Teresa Cedena, Jesús F. San-Miguel, Juan J. Lahuerta, Joan Bladé, Ruben Niesvizky, María-Victoria Mateos

**Affiliations:** 1 Hematology Department, Hospital Universitario de Salamanca (HUSAL), IBSAL, IBMCC (USAL-CSIC), CIBERONC, Salamanca, Spain; 2 Hospital Universitario de Canarias, Santa Cruz de Tenerife, Spain; 3 Hematology Department, Hospital Clinic, IDIBAPS, Barcelona, Spain; 4 Hospital Universitario de Cabueñes, Gijón, Spain; 5 Hospital Morales Meseguer, IMIB-Arrixaca, Universidad de Murcia, Murcia, Spain; 6 Institut Català d’Oncologia and Institut Josep Carreras, Hospital Germans Trias i Pujol, Barcelona, Spain; 7 Hospital Universitario de León, León, Spain; 8 Hematology Department, Catholic University of Valencia and Hospital Doctor Peset, Valencia, Spain; 9 Hospital Univeristari Vall d´Hebron, Barcelona, Spain; 10 Hospital Universitario Virgen de las Nieves, Instituto de Investigación Biosanitaria IBS GRANADA, Granada, Spain; 11 Hospital Universitario Virgen de la Victoria, Málaga, Spain; 12 Complejo Hospitalario de Navarra, Pamplona, Spain; 13 Hospital Universitario de La Princesa, Sevilla, Spain; 14 Hospital Universitario Virgen del Rocío, Sevilla, Spain; 15 Clínica Universidad de Navarra, CIMA, CIBERONC, IDISNA, Pamplona, Spain; 16 Hospital Universitario 12 de Octubre, CIBERONC, Instituto de Investigación, IMAS12 Madrid, Spain; 17 Hospital Universitario Virgen de Valme, Sevilla, Spain; 18 Hematology Department, Hospital Costa del Sol de Marbella, Marbella, Spain; 19 Hematology Department, Hospital Universitario y Politécnico La Fe, Valencia, Spain; 20 Bioaraba Health Research Institute, Oncohematology Research Group; Osakidetza, Álava University Hospital, Hematology Department, Vitoria-Gasteiz, Spain; 21 Hospital del SAS de Jerez, Jerez de la Frontera, Spain; 22 Hospital Universitario de Santiago, Santiago de Compostela, Spain; 23 Hematology Department, Hospital Universitario Marqués de Valdecilla, Santander, Spain; 24 Division of Hematology and Oncology, Weill Cornell Medicine, New York, NY USA; 25 Hospital Universitario Gregorio Marañón, Madrid, Spain; 26 CHUAC, A Coruña, Spain; 27 Hospital Clínico Universitario de Valencia, Valencia, Spain; 28 Statistics Department, University of Salamanca, Salamanca, Spain; 29 Instituto de Investigación del Hospital Universitario, 12 de Octubre, Madrid, Spain

**Keywords:** Randomized controlled trials, Myeloma

## Abstract

Although case-control analyses have suggested an additive value with the association of clarithromycin to continuous lenalidomide and dexamethasone (Rd), there are not phase III trials confirming these results. In this phase III trial, 286 patients with MM ineligible for ASCT received Rd with or without clarithromycin until disease progression or unacceptable toxicity. The primary endpoint was progression-free survival (PFS). With a median follow-up of 19 months (range, 0–54), no significant differences in the median PFS were observed between the two arms (C-Rd 23 months, Rd 29 months; HR 0.783, *p* = 0.14), despite a higher rate of complete response (CR) or better in the C-Rd group (22.6% vs 14.4%, *p* = 0.048). The most common G3–4 adverse events were neutropenia [12% vs 19%] and infections [30% vs 25%], similar between the two arms; however, the percentage of toxic deaths was higher in the C-Rd group (36/50 [72%] vs 22/40 [55%], *p* = 0.09). The addition of clarithromycin to Rd in untreated transplant ineligible MM patients does not improve PFS despite increasing the ≥CR rate due to the higher number of toxic deaths in the C-Rd arm. Side effects related to overexposure to steroids due to its delayed clearance induced by clarithromycin in this elderly population could explain these results. The trial was registered in clinicaltrials.gov with the name GEM-CLARIDEX: Ld vs BiRd and with the following identifier NCT02575144. The full trial protocol can be accessed from ClinicalTrials.gov. This study received financial support from BMS/Celgene.

## Introduction

Multiple myeloma (MM) is a tumor of clonal plasma cells producing a monoclonal immunoglobulin, and frequent devastating complications such as bone disease, hypercalcemia, renal failure, anemia, and infections. Following diagnosis, patients are usually assessed to determine whether they are eligible for high-dose chemotherapy (HDC) and autologous stem cell transplantation (ASCT). However, most of newly diagnosed MM patients are elderly or unfit and they will receive approved standard regimens until progression or the development of significant toxicity. These regimens are based on a proteasome inhibitor plus an alkylator and/or an IMiD and more recently, an anti-CD38 antibody, but always a corticosteroid is present in these combinations.

Clarithromycin is a macrolide antibiotic that through the inhibition of CYP3A4 isozyme, seems to optimize the effects of glucocorticoids by increasing the area under the curve and their maximum concentration levels^1^. In addition, clarithromycin has immunomodulatory properties, partially mediated by the suppression of interleukin-6 and other pro-inflammatory cytokines such as interleukin-1 and tumor necrosis factor α^2^. Macrolides have also been shown to modify the expression of certain cell adhesion molecules (ICAM-1, LFA antigen, and VCAM1), therefore altering the plasma cell-bone marrow stroma interactions, known to be essential in the maintenance of myeloma tumor growth^3^.

Lenalidomide plus dexamethasone (Rd) until progression is a standard of care based on the phase 3 FIRST trial in which 1623 patients with newly diagnosed MM not eligible for ASCT were randomly assigned to Rd until progression, Rd for 18 months or melphalan, prednisone and thalidomide (MPT) for 18 months. At a median follow up of 67 months, when compared with MPT, continuous Rd resulted in longer PFS (median 26 vs 22 months; hazard ratio [HR] 0.69; 95% confidence interval [CI], 0.59–0.79; *p* < 0.00001) and superior overall survival (OS) (median 59 vs 49 months; HR 0.78; 95% CI, 0.67–0.92; *p* = 0.0023)^4,5^.

In 2002, Niesvizky et al. reported an overall response rate (ORR) of 93% with the combination of clarithromycin, low-dose thalidomide and dexamethasone (C-Rd) in patients with MM in a phase 2 trial^6^. The same group published in 2013 the long-term results of the combination of C-Rd as a therapy for treatment-naïve symptomatic MM patients. After a median follow-up of 6.6 years, ORR was 93% with a very good partial response (VGPR) or better rate of 68%. Median progression-free survival (PFS) was 49 months and no increase of secondary primary malignancies was detected^7^. In a matched case-control study of 72 patients treated with C-Rd or with lenalidomide and dexamethasone (Rd) alone, the clarithromycin arm showed higher response rates, including complete response (CR) (45,8% vs 13,9%, *p* < 0.001) and VGPR or better (73.6% vs 33.3%, *p* < 0.001), as well as a higher PFS (median 48.3 vs 27.5 months, *p* = 0.044) on intention-to-treat (ITT) analysis^8^.

In this study, we have evaluated in a randomized and prospective way the efficacy and safety of clarithromycin plus lenalidomide and dexamethasone (C-Rd) as compared to Rd alone in patients with newly diagnosed MM deemed ineligible for HDT and ASCT.

## Methods

### Trial design

This multicenter, randomized, open-label, phase 3 trial enrolled patients between July 15^th^, 2015, and May 8^th^, 2019 at 20 centers in Spain selected by the Spanish Myeloma Group (GEM/PETHEMA) and at one site in the US. All patients included in the trial provided written informed consent. Independent ethics committee from each study´s site reviewed and approved the protocol, amendments, and informed consent forms. The trial was conducted in accordance with the principles of the Declaration of Helsinki and the International Conference on Harmonization-Good Clinical Practice guidelines. Dr. Niesvizky and his group at the New York Presbyterian Hospital-Cornell Medical Center designed the trial and BMS/Celgene sponsored its development. Data were compiled by the GEM/PETHEMA group.

### Patients

The trial enrolled ≥65 years old patients with newly diagnosed MM with measurable disease (M-protein in serum >0.5 g/dL, involved FLC > 10 mg/dL with abnormal κ/λ ratio, Bence Jones proteinuria >0.2 g/24 h and/or the identification of at least a plasmacytoma in a CT or MRI sized ≥1 cm in its longest diameter). Eligible patients had a Karnofsky performance status ≥60% and were unsuitable for ASCT due to age. Other inclusion criteria were to be able to receive prophylactic anticoagulation, to have a life expectancy ≥ 3 months and an absolute neutrophil count ≥1.0 ×10^9^/L, hemoglobin ≥7 g/dL, platelets ≥75.000/mm^3^ (>30.000/mm^3^ if extensive bone marrow infiltration), GOT/AST and GPT/ALT <3 times the upper limit of the normal range, and a total bilirubin level <2 mg/dL.

The trial excluded patients with unmeasurable disease, other cancers within 5 years before enrollment (except for squamous-cell and basal-cell carcinomas of the skin, carcinoma in situ of the cervix or breast and localized prostate cancer with Gleason score <7 and stable PSA), significant cardiopathy, HIV, hepatitis B and C, thromboembolic events in the last 4 weeks before inclusion or AL amyloidosis.

### Randomization and trial treatment

Eligible patients were randomly assigned in a 1:1 ratio, to receive clarithromycin plus lenalidomide and dexamethasone (C-Rd group) or lenalidomide and dexamethasone alone (Rd group).

The method used to generate the random allocation sequence was an Excel macro and the mechanism used to implement it was an interactive web response system integrated in the electronic case report form. A data manager from the GEM-PETHEMA group generated the allocation sequence; principal investigators and sub-investigators from the participant centers enrolled the patients and patients were randomly assigned to one of the two treatment arms as detailed above.

All patients received oral lenalidomide (25 mg/day on days 1 through 21) and oral dexamethasone (40 mg weekly on days 1, 8, 15, and 22) until disease progression or unacceptable toxicity. For those patients with impaired creatinine clearance, a reduced dose of lenalidomide was recommended: 10 mg/day (days 1–21) if CrCl (mL/min) was between 30 to 60, 15 mg/48 h (days 1–21) if CrCl <30 but not requiring dialysis and 5 mg/24 h (days 1–21) after the procedure in cases requiring dialysis. Patients 75 years old and older received 20 mg of weekly dexamethasone. Patients assigned to the C-Rd group received oral Clarithromycin 500 mg/12 h continuously.

### End points and assessments

The primary end point of the study was PFS, defined as the time from randomization to either disease progression or death. Secondary end points included response rates, event free survival, time to progression (TTP), OS, duration of response, PFS2, quality of life and toxicity.

TTP was defined as the time from randomization to the documented first disease progression and PFS2 as the time from randomization to the documented second disease progression. Response rates were calculated following the International Myeloma Working Group (IMWG) criteria and the overall response rate (ORR) included the sum of PR, VGPR, CR and sCR rates. Minimal residual disease (MRD) was evaluated in bone marrow aspirates by next-generation flow cytometry following the IMWG recommendations, and achieved a median limit of detection of 2 × 10^−6^ ^9^.

Urine and serum samples were obtained every 28 days (before starting a new cycle of treatment) and analyzed at the local laboratories of the participating centers to determine the presence of monoclonal proteins and the concentration of free light chains in serum. MRD was evaluated centrally in the three reference laboratories of the Spanish Myeloma Group (Madrid, Pamplona and Salamanca) and was assessed following the recommendations of the EuroFlow group in bone marrow samples obtained at the moment of achieving suspected CR and yearly afterwards^9^.

Quality of life was assessed through the FACIT fatigue scale. Toxicity analysis was based on the identification of adverse events (clinically, on physical examination and/or in laboratory or other complementary tests) graded in accordance with the NCI CTCAE (version 4).

### Statistical analysis

The primary analysis population was the intention-to-treat group of all patients who underwent randomization. The safety population comprised patients who received any dose of treatment trial. Continuous variables were analyzed with descriptive statistics, and categorical variables were summarized in frequency tables. The primary end point of PFS was compared between the treatment groups with a stratified log-rank test, and the treatment effect (HR and corresponding 95% CI) were determined by using a stratified Cox regression model, with treatment as the only explanatory variable. Other time-to-event end points were calculated similarly. Time-to-event variables were summarized with the Kaplan–Meier method. Binary endpoints, such as response rate, were assessed with a Fisher exact test, and an odds ratio and two-sided 95% CI were calculated.

Considering the results of the FIRST trial, the median PFS for patients receiving Rd was expected to be ~25.5 months. Thus, the target median PFS for patients assigned to the experimental arm was hypothesized to be 44.6 months (target hazard ratio = 1.75). To achieve 90% power to detect a 75% increase in median PFS (44.6 vs 25.5. months), at the two-sided 0.05 significance level and assuming 10% drop out rate, the required sample size was of 286.

## Results

### Patients and treatment

Between December 15th, 2015 and December 31st, 2018, a total of 286 patients were randomly assigned: 143 to the C-Rd group and 143 to the control group. Demographic and clinical characteristics of the two groups are detailed in Table 1. In the overall population, the median age was 76 years (range, 65–93), and more than half of the patients (56.6%) were 75 years of age or older.

**Table 1:** Demographic and clinical characteristics in the intention-to-treat population at baseline.

Characteristic		C-Rd Group (*n* = 143)		Rd Group (*n* = 143)
Age				
Median (range)—yr	75 (65–91)		76 (65–93)	
Distribution—no. (%)				
<75 yr	65 (45.5)		59 (41.3)	
≥75 yr	78 (54.5)		84 (58.7)	
Sex—no. (%)				
Male	71 (49.7)		64 (44.8)	
Female	72 (50.3)		79 (55.2)	
ECOG performance status—no. (%)				
0	36 (25.9)		41 (29.3)	
1	68 (48.9)		66 (47.1)	
2	33 (23.7)		29 (20.7)	
ISS disease stage—no. (%)				
I	36 (25.1)		33 (23.0)	
II	53 (37.0)		59 (41.2)	
III	54 (37.7)		51 (35.6)	
R-ISS disease stage—no. (%)				
I	15 (12.8)		17 (14.4)	
II	79 (67.5)		83 (70.3)	
III	23 (19.6)		18 (15.2)	
Type of measurable disease—no. (%)				
IgG	74 (52.1)		84 (58.7)	
IgA	47 (33)		37 (25.8)	
Bence Jones	21 (14.7)		17 (11.8)	
Cytogenetic profile—no./total no. (%)				
Standard risk	106/131 (80.9)		108/130 (83.1)	
High risk	25/131 (19.1)		22/130 (16.9)	

The intention-to-treat population was defined as all the patients who underwent randomization.

Eastern Cooperative Oncology Group (ECOG) performance status is scored from 0 to 5, with 0 reflecting no disability and higher scores indicating increasing disability.

The International Staging System (ISS) disease stage, which is obtained on the basis of the combination of serum β_2_-microglobulin and albumin levels, consists in three stages: I (β_2_-microglobulin <3.5 mg/L and albumin ≥3.5 g/dL), II (neither stage I nor stage III) and III (β_2_-microglobulin ≥ 5.5 mg/L). Higher stages indicate more advanced disease.

The R-ISS is obtained on the basis of the combination of ISS, chromosomal abnormalities (CA) detected by interphase fluorescent in situ hybridization after CD138 plasma cell purification and serum lactate dehydrogenase (LDH). R-ISS I includes ISS stage I, no high-risk CA [del(17p) and/or t(4;14) and/or t(14;16)], and normal LDH level (less than the upper limit of normal range); R-ISS III including ISS stage III and high-risk CA or high LDH level; and R-ISS II, including all other possible combinations.

Other types include IgD (one case), IgM (one case), non-secretory (two cases), and biclonal (one case).

Cytogenetic risk was based on the results of fluorescence in situ hybridization performed on CD138 + enriched bone marrow samples obtained at diagnosis.

High risk was defined by the presence of at least one of the following abnormalities: del17p, t(4;14), or t(14;16).

**Fig. 1:**
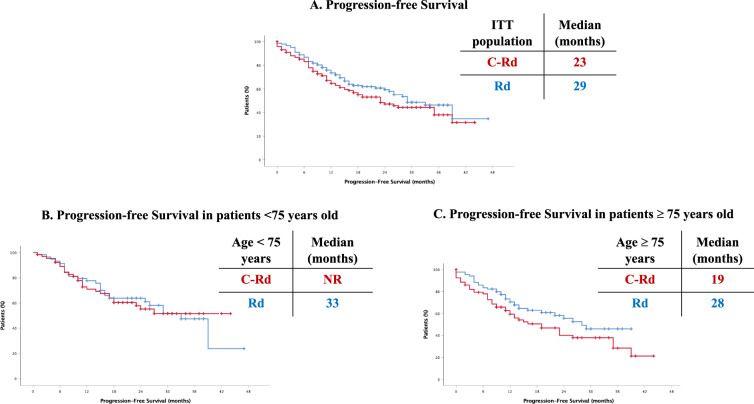
Progression-free survival. **A** In the overall cohort, (**B**) In patients <75 years old, (**C**) In patients ≥75 years old.

Among patients who were randomized, 275 (135 [94%] in the clarithromycin group and 140 [98%] in the control group) received at least one cycle of the corresponding treatment arm. At the time of the data cutoff for this analysis (February 7th, 2020), a total of 108 (75.5%) patients in the C-Rd group and 82 (57.3%) patients in the control group had discontinued treatment, most commonly due to progressive disease (31.4% in the C-Rd group and 47.5% in the control group), adverse events (25% in the C-Rd group and 19.5% in the control group) and death (22.2% in the C-Rd group and 12.2% in the control group). Individuals who discontinued treatment for reasons different than progressive disease but agreed to remain in the trial were monitored for the primary end point. A patient flow diagram has been included in the Supplementary Material (Fig. 1).

The median duration of treatment was 15.0 months (range, 0.2–44) in the C-Rd group and 15.9 months (range, 0.4–46) in the control group, and the median number of treatment cycles was 10 (range, 1–48) in the C-Rd group and 14 (range, 1–47) in the control group.

### Efficacy

At a median follow-up of 19.0 months (range, 0–54), disease progression or death was observed in 132 patients, (71 of 143 patients [49.6%] in the clarithromycin group and 61 of 143 patients [42.6%] in the control group). The Kaplan–Meier estimate of the percentage of patients who were alive without disease progression at 19 months was 53.4% (95% confidence interval [CI], 44.5–62.2) in the clarithromycin group and 61.9% (95% CI, 53.5–70.3) in the control group. The median PFS was 23 months in the clarithromycin group (95% CI 15.3–30.6) and 29 months (95% CI, 22.4–35.5) in the control group. The HR for disease progression or death in the clarithromycin group as compared to the control group was 1.293 (95% CI, 0.919–1.818, *p* = 0.14) (Fig. 1A).

Prespecified subgroup analyses of PFS (Table 2) showed that the clarithromycin group was statistically inferior as compared to the control group in patients 75 years of age or older (HR, 0.64; 95% CI, 0.41–1.0), with ISS III (HR, 0.60; 95% CI, 0.34–1.07), R-ISS III (HR, 0.26; 95% CI, 0.09–0.71); ECOG ≥2 (HR, 0.50; 95% CI, 0.29–0.88), and with a high-risk cytogenetic profile (HR, 0.46; 95% CI, 0.20–1.04).

**Table 2:** Prespecified subgroup analysis of progression-free survival.

Subgroup	Clarithromycin Group	Control Group	Clarithromycin Group	Control Group	Hazard ratio (95% CI)	
	no. of progression events or deaths/total no.		median progression-free survival (mo)			
Sex						
Male	37/71	32/64	23	26		1.24 (0.77–1.98)
Female	33/71	29/79	35	39		1.38 (0.84–2.27)
Age						
<75yr	26/64	26/59	NR	33		1.02 (0.59–1.75)
≥75yr	44/78	35/84	19	28		1.56 (1.00–2.42)
ISS						
I	10/36	11/33	NR	33		0.90 (0.38–2.13)
II	32/52	31/59	15	25		1.35 (0.82–2.20)
III	28/54	19/51	17	NR		1.67 (0.94–2.98)
Baseline creatinine clearance						
>60 ml/min	63/131	56/132	23	29		1.29 (0.90–1.84)
≤60 ml/min	7/11	5/11	9	NR		1.65 (0.52–5.20)
Type of MM						
IgG	29/74	32/84	35	39		1.06 (0.64–1.74)
Non-IgG	41/68	29/59	16	28		1.50 (0.93–2.40)
Cytogenetic profile						
High risk	17/25	9/22	12	NR		2.16 (0.96–4.86)
Standard risk	48/106	43/108	27	33		1.22 (0.81–1.84)
ECOG score						
0	11/32	18/41	39	33		0.60 (0.28–1.27)
1	30/64	22/61	26	NR		1.36 (0.79–2.36)
≥2	29/46	21/41	11	29		1.99 (1.13–3.51)
R-ISS						
I	3/15	6/17	NR	29		0.68 (0.17–2.74)
II	42/79	36/83	23	29		1.21 (0.77–1.88)
III	16/23	5/18	7	NR		3.89 (1.41–10.7)
						

Shown are the results of an analysis of progression-free survival in prespecified subgroups of the intention-to-treat population that were defined according to baseline characteristics. The experimental group was treated with clarithromycin, lenalidomide and dexamethasone; the control group received lenalidomide and dexamethasone. The International Staging System (ISS) has three stages defined as follows: I (serum β_2_-microglobulin level < 3.5 mg/L and albumin level ≥ 3.5 g/dL; stage II, neither stage I nor III; and stage III serum β_2_-microglobulin level ≥ 5.5 mg/L. A high-risk cytogenetic profile was defined by a funding of t(4;14), t(14;16), or del17p on fluorescence in situ hybridization testing. The R-ISS is obtained on the basis of the combination of ISS, chromosomal abnormalities (CA) detected by interphase fluorescent in situ hybridization after CD138 plasma cell purification and serum lactate dehydrogenase (LDH). R-ISS I includes ISS stage I, no high-risk CA [del(17p) and/or t(4;14) and/or t(14;16)], and normal LDH level (less than the upper limit of normal range); R-ISS III including ISS stage III and high-risk CA or high LDH level; and R-ISS II, including all other possible combinations. Eastern Cooperative Oncology Group (ECOG) performance status is scored on a scale from 0 to 5, with higher scores indicating increasing disability.

**Fig. 2:**
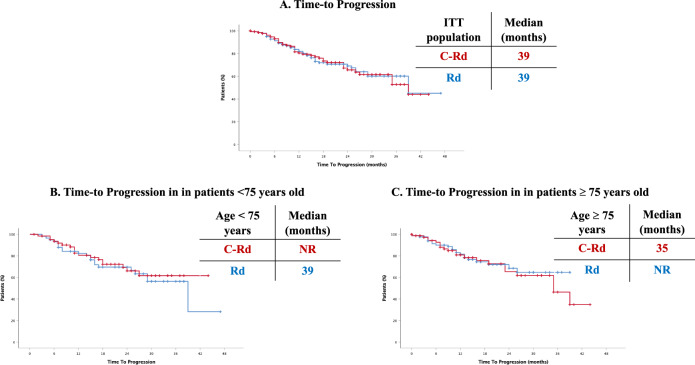
Time-to progression. **A** In the overall cohort, (**B**) In patients <75 years old, (**C**) In patients ≥75 years old.

In the ITT population, median TTP was 39 months in both arms (*p* = 0.962) (Fig. 2A). Among patients <75 years of age, median TTP was not reached in the clarithromycin group and was 39 months in the control group (*p* = 0.601) (Fig. 2B), while in ≥75 years old patients median TTP was 35 months in the clarithromycin group and not reached in the control group (*p* = 0.559) (Fig. 2C).

The percentage of patients with a CR or better was significantly higher in the clarithromycin group than in the control group (22.6% vs 14.4%, *p* = 0.048). Among 29 patients achieving CR in whom MRD was evaluated (15 from the C-Rd group and 14 from the Rd), there were no significant differences in undetectable MRD rates (4/15 [27%] of patients from the C-Rd group and in 5/14 [36%] from the Rd group). There were no statistically significant differences in the percentage of patients achieving VGPR or better (52.9% vs 46.1%) between the two groups (Table 3). The ORR was 71.5% in the clarithromycin group and 76.4% in the control group. The median time to first response was 28 days in both groups, and the median time to a CR or better was 5.5 months in the clarithromycin group and 5.4 months days in the control group.

**Table 3:** Summary of response rates.

	C-Rd Group (*n* = 143)	Rd Group (*n* = 143)	*P* value
Overall response—no. (%)	71.5	76.4	
Best overall response – no (%)			
Complete response or better	33 (22.6)	21 (14.4)	0.048
Stringent complete response	31 (21.3)	18 (12.4)	0.029
Complete response	2 (1.3)	3 (2.0)	0.500
Very good partial response or better	77 (52.9)	67 (46.1)	0.144
Very good partial response	44 (30.3)	46 (31.7)	0.449
Partial response	27 (18.6)	44 (30.3)	0.014
Stable disease	27 (18.6)	31 (21.3)	0.330
Progressive disease	1 (0.6)	1 (0.6)	0.751
Response could not be evaluated	0 (0)	3 (2.0)	0.124

Response was assessed following the guidelines of the International Myeloma Working Group. The P value was calculated with the use of the Fisher´s exact test.

**Fig. 3:**
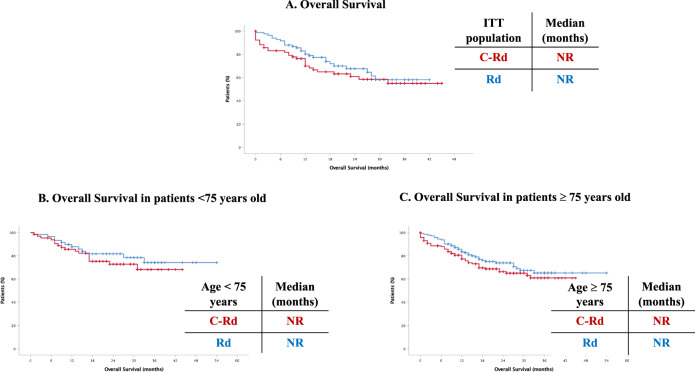
Overall survival. **A** In the overall cohort, (**B**) In patients <75 years old, (**C**) In patients ≥75 years old.

At a median follow-up of 19 months, 84 patients had died, 46 (32.1%) in the clarithromycin group and 38 (26.5%) in the control group. The median OS has not been reached yet in either group (Fig. 3A–C).

### Safety

Table 4 summarizes the most common adverse events (AEs) of any grade (Table 4a) and the most common AEs of grade 3 or 4 (Table 4b) during treatment in the safety population, globally and across age groups. The most common AEs of grade 3 or 4 were neutropenia (12% vs 19% in de C-Rd and Rd groups, respectively [p = ns]), asthenia (11% vs 3%, [*p* = 0.009]) and steroid-related (15% vs 6%, [*p* = 0.021]). Among steroid-related adverse events, we included tremors, anxiety, insomnia, nervousness, myopathy, hyperglycemia, confusion, cataracts, facial edema, and aphonia. Overall, the incidence of infections of any grade was 54%; the incidence of grade 3 or 4 infections was 30% in the clarithromycin group and 25% in the control group (*p* = ns). When we analyzed toxicity dividing the cohort by age, we found out that among patients ≤75 years old, grade 3–4 adverse events were almost equally reported in the two arms; in contrast, among patients >75 years old, the rates of asthenia (14% vs 3% in C-Rd and Rd respectively, [*p* = 0.024]), steroid-related AEs (19% vs 5%, [*p* = 0.006]) and infections (33% vs 25%, [*p* = ns]) were higher in the clarithromycin group with neutropenia being more frequently identified in the Rd group (14% vs 25%, [*p* = ns]).

**Table 4:** a Most common adverse events and second primary malignancies reported during treatment in the safety population. b Most common adverse events of grade 3–4 reported during treatment in the safety population.

	No. (%)								
	All			Age ≤ 75 Years			Age >75 Years		
TEAE or SPM	C-Rd (*n* = 143)	Rd (*n* = 143)	*p*	C-Rd (*n* = 65)	Rd (*n* = 59)	*p*	C-Rd (*n* = 78)	Rd (*n* = 84)	*p*
a									
Hematologic TEAEs, any grade									
Neutropenia	34 (24)	51 (36)	0.038	15 (23)	14 (24)	1	19 (24)	37 (44)	0.013
Anemia	26 (18)	28 (20)	0.880	7 (14)	8 (14)	0.784	19 (24)	20 (24)	1
Thrombocytopenia	21 (15)	22 (15)	1	9 (14)	7 (12)	0.794	12 (15)	15 (18)	0.833
Nonhematologic TEAEs, any grade									
Astenia	40 (28)	44 (31)	0.697	17 (26)	12 (20)	0.526	23 (29)	32 (38)	0.319
Steroid-related	58 (41)	40 (28)	0.034	28 (34)	17 (29)	0.134	30 (38)	23 (27)	0.180
Infections	78 (55)	76 (53)	0.906	32 (49)	28 (47)	0.859	46 (59)	48 (57)	0.874
Pneumonia	11 (8)	19 (13)	0.176	6 (9)	7 (12)	0.771	5 (6)	12 (14)	0.127
DVT and EP	9 (6)	11 (8)	0.817	5 (8)	5 (8)	1	4 (5)	6 (7)	0.748
Diarrhea	22 (15)	24 (17)	0.872	10 (15)	10 (17)	1	12 (15)	14 (17)	0.834
Invasive second primary cancer	4 (3)	1 (1)	0.371	1.(1)	0 (0)	1	3 (4)	1 (1)	0.353

b									
Hematologic TEAEs, any grade									
Neutropenia	17 (12)	28 (19)	0.104	6 (9)	7 (12)	0.771	11 (14)	21 (25)	0.114
Anemia	4 (3)	10 (7)	0.169	1 (2)	5 (8)	0.101	3 (4)	5 (6)	0.721
Thrombocytopenia	7 (5)	4 (3)	0.541	2 (3)	1 (2)	1	5 (6)	3 (3)	0.483
Nonhematologic TEAEs, any grade									
Astenia	16 (11)	4 (3)	0.009	5 (8)	1 (2)	0.211	11 (14)	3 (3)	0.024
Steroid-related	22 (15)	9 (6)	0.021	7 (11)	5 (8)	0.766	15 (19)	4 (5)	0.006
Infections	43 (30)	36 (25)	0.428	17 (26)	15 (25)	1	26 (33)	21 (25)	0.299
Pneumonia	10 (7)	14 (10)	0.523	6 (9)	6 (10)	1	4 (5)	8 (9)	0.373
DVT and EP	2 (1)	6 (4)	0.282	0 (0)	4 (7)	0.049	2 (2)	2 (2)	1
Diarrhea	4 (3)	5 (3)	1	2 (3)	2 (3)	1	2 (2)	3 (3)	1
Invasive second primary cancer	4 (3)	1 (1)	0.371	1 (1)	0 (0)	1	3 (4)	1 (1)	0.353

Serious AEs were reported in 55.9% of the patients in the clarithromycin group and in 47.6% of the patients in the control group. The most common serious AEs in both groups were infectious, 24% in the clarithromycin group and 28% in the control group. The percentage of patients who developed adverse events leading to discontinuation of the trial treatment was 25% in the C-Rd group and 19.5% in the control group. Trial discontinuation due to infection occurred in 1 patient in each group.

Adverse events resulting in death were observed in 36 patients (25%) in the clarithromycin group and in 22 patients (15%) in the control group. The most frequent adverse events were infections, which resulted in death in 10% (14/143) and 5% (7/143) of the patients, respectively. Invasive second primary cancers occurred in 4 patients (3%) in the clarithromycin group (solid tumors in 4 cases [2%] and hematologic cancers in 1 case [1%]) and in 1 patient (1%) in the control group (solid tumor).

The median relative dose intensity for clarithromycin was 80% (Table 5). The median relative dose intensity for lenalidomide was 72.1% in the clarithromycin group and 83.3% in the control group; 14 patients (9.7%) in the clarithromycin group and 19 patients (13.2%) in the control group received 10 mg or less as a starting dose of lenalidomide. The percentage of patients that required dose modifications of lenalidomide (including lenalidomide discontinuations) were 54.5% in the clarithromycin group and 51% in the control group; as per dexamethasone, the percentage of patients that required dose modifications (including discontinuations) were 49.6% in the clarithromycin group *vs* 46.5% in the control group. One patient discontinued lenalidomide in the clarithromycin group; 6 patients in the clarithromycin group and 4 in the control group discontinued dexamethasone and 16 patients discontinued clarithromycin. The median relative dose intensity for dexamethasone was 62.8% in the clarithromycin group and 84.3% in the control group.

**Table 5:** Treatment exposure in the safety population.

	C-Rd Group (*n* = 143)	Control Group (*n* = 143)
Median duration of study treatment—mo (range)	15 (0.2–44)	15.9 (0.4–46)
Median total number of cycles received—no. (range)	10 (1–48)	14 (1–47)
Median relative dose intensity—% (range)		
Clarithromycin	80 (35.7–98.4)	–
Lenalidomide	72.1 (0–98.6)	83.3 (19–97.0)
Dexamethasone	62.8 (0–71.4)	84.3 (0–88.7)

## Discussion

Retrospective and single-arm studies performed in relapsed/refractory and newly diagnosed MM patients reported better outcomes when clarithromycin was combined with immunomodulatory drugs, either thalidomide, lenalidomide and pomalidomide. In this phase 3 trial for patients with newly diagnosed MM who were ineligible for HDT and ASCT, the combination of clarithromycin, lenalidomide and dexamethasone resulted in a rate of CR or better significantly higher than that obtained with lenalidomide and dexamethasone alone (22.6% vs 14.4%, *p* = 0.048); however, the observed median PFS was not significantly different between the two groups of patients (23 months in the C-Rd arm vs 29 months in the Rd arm).

To contextualize our results, the median PFS observed in the control group was comparable to that reported by other studies performed in newly diagnosed MM patients ineligible for ASCT.^4,10–13^. In the FIRST, MAIA and SWOG trials, the median PFS reached by the group of patients receiving lenalidomide and dexamethasone was 26.0, 34.4 and 28.9 months, respectively. Importantly, the percentage of patients 75 years of age or older in our study (56.6%) was higher than the reported in these three trials (43%, 43% and 48% ≥65 years old in each of them, respectively).

Furthermore, the ORR observed in the control arm of our study (76.4%) is comparable to what has been reported in the above trials with continuous Rd as control arm (FIRST trial, 75%, MAIA, 82% and SWOG, 78.8%)^4,10–13^.

However, the addition of clarithromycin to the standard Rd did not result into a significant benefit in PFS in our trial. This contrasts with previous retrospective and single-arm studies performed in relapsed/refractory and newly diagnosed MM patients reporting better outcomes when clarithromycin was combined with immunomodulatory drugs, either thalidomide, lenalidomide and pomalidomide. Niesvizky et al. published in 2013 the long-term results of C-Rd in NDMM patients^7^. After a median follow-up of 6.6 years, ORR was 93% with a VGPR or better rate of 68% and a median PFS of 49 months. In our study, the ORR rates were also comparable in both arms although the percentage of patients with a CR or better was significantly higher in the clarithromycin group than in the control group (22.6% vs 14.4%, *p* = 0.048)^7^. This can be due to the increased activity achieved by adding clarithromycin to Rd as it has been previously described in the setting of newly diagnosed MM using the same schedule^8^. Furthermore, a phase 2 trial evaluating the safety and efficacy of clarithromycin, pomalidomide and dexamethasone in relapsed or refractory MM reported an ORR of 60% (23% ≥VGPR), which is significantly higher than that reported in patients treated with Pomalidomide and dexamethasone alone in the MM-002 study (32.7%)^14^, in MM-003 (31.4%)^15^, in STRATUS (32.6%)^16^, and in IFM- 2009-02 (34.5%)^17^.

Importantly, as stated above, more than half of the patients in our trial were ≥75 years old. In depth analysis of our results showed that, whereas in patients <75 years old, PFS was comparable in both arms (NR, 33 months; *p* = ns), in the group of patients 75 years old and older, median PFS was shorter in the C-Rd group (19 vs 28 months; *p* = 0.05) (Fig. 1B-C). TTP was then analyzed as a secondary endpoint: no differences were observed when analyzing the ITT population and similar results were obtained in the group of patients <75 years (NR in C-Rd and 39 months in Rd) and in those ≥75 years old (35 months in C-Rd vs NR in Rd). Thus, in the ≥75 years old cohort, PFS was longer in the control group with no differences in TTP between the two arms, thus suggesting that the higher treatment-related mortality in these older patients could be explaining our results.

We then analyzed the 90 documented deaths, 50 in the C-Rd group and 40 in the Rd group. Whereas the number of patients dying in the context of PD was similar in both groups (14 patients in the C-Rd group and 18 in the Rd group), there were 36 cases in the C-Rd arm vs 22 in the Rd arm who died in the absence of PD (14 *vs* 7 due to infections, 5 vs 3 of cardiovascular origin and 3 vs 0 due to secondary primary malignancies, respectively; in 8 cases in each group, the exact cause of death was unknown). The percentage of patients ≥75 years old dying without associated PD was 52% (26/50) in the C-Rd arm and 40% (16/40) in the Rd arm. Therefore, a higher number of patients ≥75 years old dying without associated PD in the C-Rd arm (26 vs 16 in the Rd arm) was responsible for the shorter PFS observed in this group.

When we analyzed the most common AE of grade 3–4, neutropenia and infections were the most frequently reported in both arms (19% and 25% [10% of pneumonia] in Rd vs 12% and 30% [7% pneumonia] in C-Rd, respectively). As compared to the Rd arm, a slightly higher percentage of cases presented with infections in the C-Rd arm, but also with more frequent asthenia (11% vs 3%) and steroid-related AEs (15% vs 6%). The most frequent AEs reported in the group of patients receiving Rd in the MAIA and FIRST trials were neutropenia and infections, as presented in the Rd arm in our study. On the other hand, the most common grade 3 or higher nonhematologic adverse events reported in the BiRd trial included myopathy (11.1%), neuromood (4.2%) and tremor (4.2%), all of them attributable to steroids and also more frequently developed by the C-Rd arm in our study^18^. Similarly, in the ClaPd trial for relapsed and refractory MM patients, the most common nonhematologic grade 3 or higher toxicities were fatigue (15%), pulmonary infection (13%), and hyperglycemia (15%)^3^. Importantly, the median age in these two studies was significantly lower than in ours: 63 (range, 36–83) in the BiRd trial, 63 (range, 42–87) in the ClaPd trial and 76 (range, 65–93) in our trial.^3,18^. In fact, whereas among patients ≤ 75 years old, we found that grade 3–4 adverse events were almost equally reported in the two arms, in patients >75 years old, the rates of asthenia (14% vs 3% in C-Rd and Rd respectively), steroid-related AEs (19% vs 5%) and infections (33% vs 25%) were higher in the clarithromycin group.

The median treatment duration was similar in the two groups (15.0 vs 15.9 months) but, the median number of treatment cycles was lower in the C-Rd group vs the Rd group (10 vs 14 months). Besides, due to the higher incidence of grade 3–4 AEs, patients in the C-Rd group received less lenalidomide (median relative dose intensity of 72.1% vs 83.3%) and less dexamethasone (median relative dose intensity of 62.8% vs 84.3%) than patients treated with Rd. Thus, the similar PFS between the two groups in our study might also be related to the lower treatment compliance observed in the clarithromycin group.

Recent trials have explored the clinical impact of sparing steroids in elderly patients with MM. In the study presented by Larocca et al. at the EHA2020, 199 intermediate-fit patients were randomized 1:1 to receive standard continuous Rd or 9 cycles of Rd followed by maintenance with lenalidomide (10 mg po od days 1–21), both until progressive disease or intolerance. Best response rates seemed to favor the experimental arm (≥ nCR 19% vs 15% and ≥ VGPR 44% vs 35% in Rd-R vs Rd, respectively) and no differences in PFS (43% and 42% at 20 months in Rd-R vs Rd) and OS (84% and 79% at 20 months in Rd-R vs Rd) were observed between the two groups. However, patients assigned to the continuous Rd treatment presented with more grade 3 or higher non-hematological toxicities, higher discontinuation rates and higher dose reductions due to AEs^19^. Similarly, in the updated results of the EMN01 randomized trial published by Bringhen et al., the authors evaluated maintenance treatment with lenalidomide with or without prednisone in 402 patients previously treated with Rd and melphalan or cyclophosphamide as induction. From the start of maintenance, there were no differences in PFS between patients treated with lenalidomide-prednisone vs those receiving lenalidomide alone (22.2 vs 18.8 months, HR 0.85, *p* = 0.14), with no differences across frailty subgroups defined as per the IMWG Frailty Score^20^.Thus, in this group of elderly patients with MM, steroids could be reduced or avoided without compromising treatment efficacy of lenalidomide. Also, in steroid sparing regimens, clarithromycin could be added to test its direct immunomodulatory and anti-tumor properties, but avoiding the side effects derived from its influence on the kinetics of glucocorticoids.

In conclusion, the addition of clarithromycin to Rd in transplant ineligible newly diagnosed MM patients is not associated with an improved PFS, despite a significantly increase in the CR rate as compared to Rd; this is due to a higher proportion of toxic deaths in the C-Rd arm, mostly infectious and concentrated in the group ≥75 years old, which accounted for half of the recruited patients. In this elderly population, overexposure to steroids due to the delayed clearance induced by clarithromycin together with a lower treatment compliance could explain our results. Further investigations modifying the dose of both clarithromycin and steroids based on age and frailty status could be of interest to exploit the benefits of this combination. The results of our trial also highlight the relevance of evaluating phase I/II clinical data in the context of phase III clinical trials, since such design offers us the strongest scientific evidence regarding efficacy and safety of new treatments as compared to the standard of care.

## Supplementary information

Patient flow diagram

Supplementary material

CLARIDEX_CONSORT

CLARIDEX_Checklist

## References

[1] Spahn JD (2001). Clarithromycin potentiates glucocorticoid responsiveness in patients with asthma: Results of a pilot study. Ann. Allergy Asthma Immunol..

[2] Ohara T (2004). Antibiotics directly induce apoptosis in B cell lymphoma cells derived from BALB/c mice. Anticancer Res.

[3] Mark TM (2019). Phase 2 study of clarithromycin, pomalidomide, and dexamethasone in relapsed or refractory multiple myeloma. Blood Adv.

[4] Benboubker L (2014). Lenalidomide and dexamethasone in transplant-ineligible patients with myeloma. N. Engl. J. Med..

[5] Facon T (2018). Final analysis of survival outcomes in the phase 3 FIRST trial of up-front treatment for multiple myeloma. Blood..

[6] Coleman M (2002). BLT-D (clarithromycin [Biaxin], low-dose thalidomide, and dexamethasone) for the treatment of myeloma and Waldenström’s macroglobulinemia. Leuk. Lymphoma.

[7] Rossi A (2013). BiRd (clarithromycin, lenalidomide, dexamethasone): An update on long-term lenalidomide therapy in previously untreated patients with multiple myeloma. Blood..

[8] Gay F (2010). Clarithromycin (Biaxin)-lenalidomide-low-dose dexamethasone (BiRd) versus lenalidomide-low-dose dexamethasone (Rd) for newly diagnosed myeloma. Am. J. Hematol..

[9] Flores-Montero J (2017). Next Generation Flow for highly sensitive and standardized detection of minimal residual disease in multiple myeloma. Leukemia..

[10] Facon T (2019). Daratumumab plus lenalidomide and dexamethasone for untreated myeloma. N. Engl. J. Med..

[11] Durie PBGM (2017). Bortezomib with lenalidomide and dexamethasone versus lenalidomide and dexamethasone alone in patients with newly diagnosed myeloma without intent for immediate autologous stem-cell transplant (SWOG S0777): a randomised, open-label, phase 3 trial. Lancet.

[12] Durie B. G. M. et al. Longer term follow-up of the randomized phase III trial SWOG S0777: bortezomib, lenalidomide and dexamethasone vs. lenalidomide and dexamethasone in patients (Pts) with previously untreated multiple myeloma without an intent for immediate autologous stem cell transplant (ASCT). Blood Cancer J. [Internet]. 2020;10. 10.1038/s41408-020-0311-8 PMC7214419 32393732

[13] Kumar S. K. et al. Updated analysis of daratumumab plus lenalidomide and dexamethasone (D-Rd) versus lenalidomide and dexamethasone (Rd) in patients with transplant-ineligible newly diagnosed multiple myeloma (NDMM): the phase 3 Maia study. Blood (2020) 136 (Supplement 1): 24–26. Abstract 2276.

[14] Richardson PG (2014). Pomalidomide alone or in combination with low-dose dexamethasone in relapsed and refractory multiple myeloma: a randomized phase 2 study. Blood [Internet].

[15] Miguel JS (2013). Pomalidomide plus low-dose dexamethasone versus high-dose dexamethasone alone for patients with relapsed and refractory multiple myeloma (MM-003): a randomised, open-label, phase 3 trial. Lancet Oncol. [Internet].

[16] Dimopoulos MA (2016). Safety and efficacy of pomalidomide plus low-dose dexamethasone in STRATUS (MM-010): a phase 3b study in refractory multiple myeloma. Blood [Internet].

[17] Leleu X (2013). Pomalidomide plus low-dose dexamethasone is active and well tolerated in bortezomib and lenalidomide–refractory multiple myeloma: Intergroupe Francophone du Myélome 2009-02. Blood.

[18] Niesvizky R (2008). BiRD (Biaxin [clarithromycin]/Revlimid [lenalidomide]/dexamethasone) combination therapy results in high complete- and overall-response rates in treatment-naive symptomatic multiple myeloma. Blood..

[19] Larocca A. et al. Sparing steroids in elderly intermediate-fit newly diagnosed multiple myeloma patients treated with a dose/schedule-adjusted Rd-R vs. continuous Rd: results of RV-MM-PI-0752 phase III randomized study. HemaSphere 2019; 3(S1): 244. Abstract PF586.

[20] Bringhen S (2020). Lenalidomide-based induction and maintenance in elderly newly diagnosed multiple myeloma patients: updated results of the EMN01 randomized trial. Haematologica.

