# Supplementary figures and images for: Lenalidomide and dexamethasone with or without clarithromycin in patients with multiple myeloma ineligible for autologous transplant: a randomized trial
Source: Blood Cancer J. 2021 May 21;11(5):101. doi: 10.1038/s41408-021-00490-8 (PMC8139975; doi:10.1038/s41408-021-00490-8)

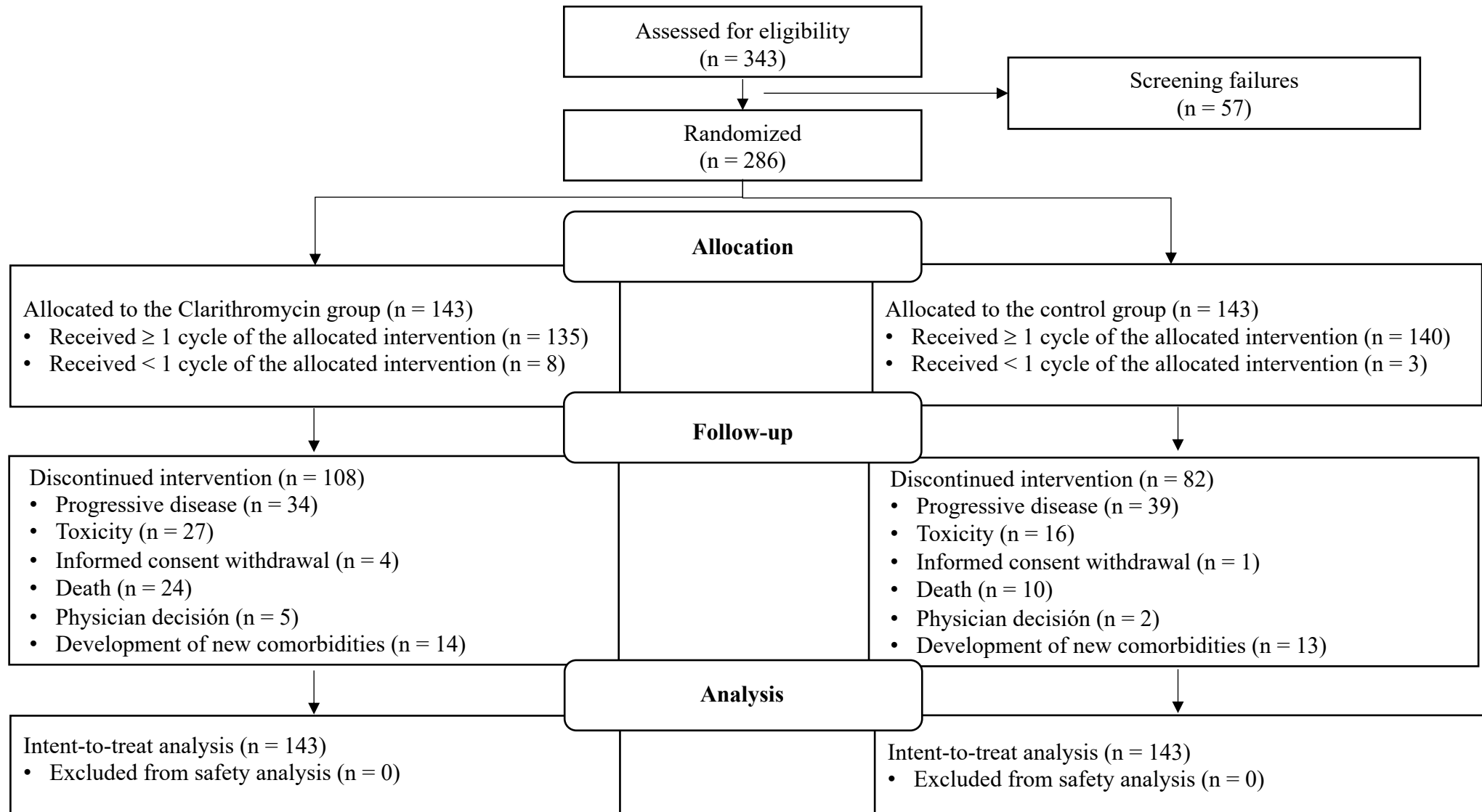

Supplement: Supplementary file 1 — Patient flow diagram [file 41408_2021_490_MOESM1_ESM.pdf]
