# Supplementary material for: Lenalidomide and dexamethasone with or without clarithromycin in patients with multiple myeloma ineligible for autologous transplant: a randomized trial
Source: Blood Cancer J. 2021 May 21;11(5):101. doi: 10.1038/s41408-021-00490-8 (PMC8139975; doi:10.1038/s41408-021-00490-8)
Supplement: Supplementary file 2 — Supplementary material [file 41408_2021_490_MOESM2_ESM.docx]

Figure Legends.

Patient Flow Diagram

**List of the Investigators in the GEM (Grupo Español de Mieloma)/PETHEMA (Programa para el Estudio de la Terapéutica en Hemopatías Malignas) Cooperative Study Group**

Dr. Maria Casanova Espinosa, Complejo Hospitalario Costa del Sol; Dr. José Luis Guzmán Zamudio y Dr. Sebastián Garzón, H. Especialidades de Jerez de la Frontera; Dr. Eduardo Ríos Herranz y Dr. María del Carmen Couto, H. Nuestra Señora de Valme; Dr. Rafael Ríos Tamayo, H. Universitario Virgen de las Nieves; Dr. Jesús Martín Sánchez, Complejo Hospitalario Regional Virgen del Rocío; Dr. Luis Palomera Bernal, H. Clínico Universitario Lozano Blesa; Dr. Ana Pilar González Rodríguez, H. Universitario Central de Asturias; Dr. María Esther González García, H. Cabueñes; Dr. Antonia Sampol Mayol, Complejo Asistencial Son Espases; Dr. Joan Bargay Lleonart, H. Son Llátzer; Dr. Alexia Suárez, H. de Gran Canaria Dr. Negrín; Dr. Miguel Teodoro Hernández García, H. Universitario de Canarias; Dr. Carmen Montes Gaisán y Guillermo Martín-Sánchez, H. Universitario Marqués de Valdecilla; Dr. Belén Hernández Ruiz, H. General de Ciudad Real; Dr. Felipe Casado Montero, Complejo Hospitalario de Toledo; Dr. Dunia de Miguel Llorente, H. Universitario de Guadalajara; Dr. Fernando Solano Ramos, H. Nuestra Señora del Prado (Talavera); Dr. Ángela Ibáñez García, H. General de Albacete; Dr. Noemi Puig, Dr. Verónica González-Calle, Dr. Enrique M. Ocio, Dr. Norma C. Gutiérrez and Dr. María-Victoria Mateos, Hospital Universitario de Salamanca, H. Clínico de Salamanca; Dra. Aránzazu García-Mateo, Complejo Hospitalario H. General de Segovia; Dr. Fernando Escalante Barrigón, H. de León; Dr. Javier García Frade, H. Universitario Rio Hortega; Dr. Alfonso García de Coca, H. Clínico Universitario de Valladolid; Dr. Carlos Aguilar Franco, H. Santa Bárbara; Dr. Jorge Labrador Gómez, Hospital Universitario de Burgos; Dr. Elena Cabezudo Pérez, H. Althaia, Xarxa Asistencial de Manresa (Sant Joan de Deu); Dr. Joan Bladé Creixentí, Hospital Clinic de Barcelona; Dr. Ana María Sureda Balari, H. Durán i Reynals—ICO L´Hospitalet; Dr. Yolanda González Montes, ICO Girona, H. Universitario de Girona Dr. Josep Trueta; Dr. Lourdes Escoda Teigell, H. Universitario Joan XXIII de Tarragona; Dr. Antonio García Guiñón, Hospital Universitari Arnau de Vilanova de Lleida; Dr. Eugenia Abella Monreal, H. del Mar; Dr. Juan Alfonso Soler Campos, H. de Sabadell (Parc Taulí); Dr. Josep Maria Martí Tutusaus, Hospital Universitario Mutua de Terrassa; Dr. Albert Oriol Rocafiguera, H. Germans Trias i Pujol; Dr. Miquel Granell Gorrochategui, H. de la Santa Creu i Sant Pau; Dr. Mercedes Gironella Mesa, H. Vall d´Hebrón; Dr. Carmen Cabrera Silva, H. San Pedro de Alcántara (Complejo Hospitalario de Cáceres); Dr. Marta Sonia González Pérez, Complejo Hospitalario Universitario de Santiago; Dr. Ana Dios Loureiro, Complejo Hospitalario de Pontevedra; José Angel Méndez Sánchez, Complejo Hospitalario de Ourense; Dr. María Josefa Nájera Irazu, H. San Pedro; Dr. Francisco Javier Peñalver Párraga, H. Universitario Fundación de Alcorcón; Dr. Juan José Lahuerta Palacios and Dr. M. Teresa Cedena, H. Universitario 12 de Octubre; Dr. Pilar Bravo Barahona, H. de Fuenlabrada; Dr. Cristina Encinas Rodríguez, H. General Universitario Gregorio Marañón; Dr. José Ángel Hernández Rivas, H. Infanta Leonor; Dr. Jaime Pérez de Oteyza, H. Universitario Madrid—Sanchinarro; Dr. Rebeca Iglesias del Barrio, Centro Oncológico MD Anderson; Dr. Ana López de la Guía, H. Universitario La Paz; Dr. Adrián Alegre Amor, H. Universitario de la Princesa; Dr. Elena Prieto Pareja, Fundación Jiménez Díaz—UTE; Dr. Isabel Krsnik Castelló, Hospital Universitario Puerta de Hierro—Majadahonda; Dr. María Jesús Blanchard Rodríguez, H. Ramón y Cajal; Dr. Rafael Martínez, H. Universitario de San Carlos; Dr. Rosalía Riaza Grau, H. Severo Ochoa; Dr. Eugenio Giménez Mesa, H. Infanta Sofía; Dr. Elena Ruiz Sainz, Hospital del Tajo; Dr. Felipe de Arriba, H. Morales Meseguer; Dr. José María Moraleda Jiménez, H. Universitario Virgen de la Arrixaca; Dr. Marta Romera, H. General Universitario Santa Lucia; Dr. Felipe Prósper Cardoso, Clínica Universidad de Navarra; Dr. José María Arguiñano Pérez, Complejo Hospitalario de Navarra; Dr. María Puente Pomposo, H. de Cruces; Dr. Ernesto Pérez Persona, H. de Txagorritxu; Dr. Ana Isabel Teruel Casasús, H. Clínico Universitario de Valencia; Dr. Paz Ribas García and Dr. Javier de la Rubia, H. Universitario Dr. Peset; Dr. Mario Arnao, H. Universitario La Fe; Dr. María Blanca Villarrubia Lor, H. General Universitario de Alicante; Dr. Pedro Luis Fernández García, H. Torrevieja; Dr. Ricarda García-Sánchez, Hospital Universitario Virgen de la Victoria, Málaga; Dr. Laura Rosiñol, Hospital Clinic de Barcelona; Dr. Ana M. Vale, CHUAC, La Coruña; Dr. María J. Calasanz, Dr. Bruno Paiva and Dr. Jesús F. San Miguel, Clínica Universidad de Navarra, Pamplona.
